# Procedural Theatre: EU-HTA Between Ritual and Purpose—Why the Buyer’s Audit Has Outgrown the Transaction

**DOI:** 10.3390/jmahp14030052

**Published:** 2026-08-29

**Authors:** Mondher Toumi, Maarten Jacobus Postma, Bruno Falissard, Frank-Ulrich Fricke, Stefano Capri, Jürgen Wasem, Steven Simoens, Renato Bernardini, Malgorzata Wojtal, Anna Kapuśniak, Oriol Sola Morales, Laurent Boyer, Jaime Espin, Pascal Auquier

**Affiliations:** 1 CEReSS/UR3279—Health Services Research and Quality of Life Center, Aix-Marseille University, 13385 Marseille, France; laurent.boyer@univ-amu.fr (L.B.); pascal.auquier@univ-amu.fr (P.A.); 2 Department of Health Sciences, University Medical Center Groningen, University of Groningen, 9700 AB Groningen, The Netherlands; postmamj57@gmail.com; 3 Department of Economics, Econometrics & Finance, Faculty of Economics & Business, University of Groningen, 9713 AB Groningen, The Netherlands; 4 Center of Excellence in Higher Education for Pharmaceutical Care Innovation, Universitas Padjadjaran, Bandung 40132, Indonesia; 5 Division of Pharmacology & Therapy, Faculty of Medicine, Universitas Airlangga, Surabaya 60131, Indonesia; 6 CESP, INSERM U1018, Université Paris-Saclay, 94800 Villejuif, France; bruno.falissard@gmail.com; 7 Fachbereich Betriebswirtschaft, Technische Hochschule Nürnberg, 90402 Nürnberg, Germany; frank-ulrich.fricke@th-nuernberg.de; 8 School of Economics and Management, Cattaneo-LIUC University, 21053 Castellanza, Italy; scapri@liuc.it; 9 Institute of Healthcare Management, Universität Duisburg-Essen, 45127 Essen, Germany; juergen.wasem@medman.uni-due.de; 10 Department of Pharmaceutical and Pharmacological Sciences, Katholieke Universiteit Leuven, 3000 Leuven, Belgium; steven.simoens@kuleuven.be; 11 Section of Pharmacology, Department of Biomedical and Biotechnological Sciences (BIOMETEC), University of Catania, 95124 Catania, Italy; bernardi@unict.it; 12 Clever-Access, 30-415 Krakow, Poland; malgorzata.wojtal@clever-access.com (M.W.); anna.kapusniak@clever-access.com (A.K.); 13 HiTT Foundation, 08015 Barcelona, Spain; osola@hittbcn.com; 14 Andalusian School of Public Health/Escuela Andaluza de Salud Pública (EASP), 18011 Granada, Spain; jaime.net2010@gmail.com; 15 CIBER of Epidemiology and Public Health (CIBERESP), 28029 Madrid, Spain; 16 Instituto de Investigación Biosanitaria, 18012 Granada, Spain

## 1. Introduction

Health technology assessment is a discipline that has developed, over five decades, an elaborate scientific and institutional identity centered on the evaluation of clinical evidence, comparative effectiveness, and economic value. Yet behind this intellectual architecture lies an elementary transaction: a manufacturer seeks to sell a medicine, a payer seeks to buy it, and a price must be agreed upon that reflects both the value of the product and the buyer’s willingness and ability to pay to secure the reimbursement. Every formal process that occurs between regulatory approval and patient access, coverage decision, benefit assessment, cost-effectiveness analysis, and managed entry negotiation is, in the final analysis, an instrument for arriving at, or contesting, a price [1,2]. Demand and supply could not set the price, as the buyer (prescriber), the payer (insurer), and the consumer (patient) are different individuals, unlike standard goods like a car or a mobile phone, for example. The underlying reason is market failure: a medicine funded by the public payer rather than the patient escapes ordinary supply-and-demand price formation, so a pricing and reimbursement mechanism must determine the price at which it is worth funding [3].

This assertion is not a reductionist critique of the field; it is an epistemological clarification that the field itself tends to suppress. In the United Kingdom, NICE (National Institute for Health and Care Excellence) reimbursement recommendations function as de facto price signals: a negative recommendation precipitates a price reduction by the manufacturer to cross the £20,000–£30,000/QALY threshold, rendering the binary coverage framing an institutional euphemism for a price negotiation [4,5]. In Germany, the G-BA benefit rating directly determines the price range achievable in subsequent negotiations with the GKV-Spitzenverband [6]. In France, the HAS Commission de la Transparence explicitly assigns both a reimbursement rate and an ASMR rating that governs CEPS pricing negotiations, making the dual function of HTA legally transparent [7,8,9]. France is the only country that disconnects technically the price driver from the reimbursement recommendation. However, reimbursement is only applied if a price is agreed upon. Across jurisdictions, the institutional form varies; the economic substance is invariant [10,11].

It is against this backdrop that the EU Joint Clinical Assessment (JCA), established under Regulation (EU) 2021/2282, must be evaluated [12]. The JCA constitutes, formally, a clinical rather than an economic assessment; it does not set prices, does not determine reimbursement, and does not replace national HTA. Yet, its practical function is to supply the shared evidentiary input upon which national pricing and access decisions will be made across all member states. Understanding it as a price-determination input and asking whether its procedural architecture is proportionate to that function is, therefore, not only legitimate but necessary.

## 2. HTA as the Buyer’s Audit of Product Specification

All commerce requires two parties to agree on a price and a specification. The seller must communicate what the product does; the buyer must determine what it is worth. In pharmaceutical markets, the product specification is inherently probabilistic: at the time of launch, the evidence base is largely limited to randomized controlled trials, which necessarily characterize clinical benefit within restricted populations, under protocol-driven patient management, and over time horizons shorter than those over which the product will ultimately be used. HTA is the institutional mechanism through which the payer audits that specification: it scrutinizes the evidence, contextualizes the comparators, characterizes the uncertainty, and translates clinical findings into a value framework that informs the price the payer is willing to accept [7,13].

This audit function is legitimate and important. A payer that simply accepts a manufacturer’s price without independent assessment of clinical value would be commercially and institutionally negligent. HTA fills this role, and its contribution to rational resource allocation in health systems is well-documented [14,15]. The question is not whether HTA should exist but whether the scale and depth of the audit should be proportionate to the commercial transaction it is designed to support and whether that proportionality requirement has been respected in the EU-HTA architecture.

The logic of proportionality in commercial transactions is robust: disproportionate audit costs are borne by both parties, delay the transaction, and do not necessarily improve its terms. Or, to phrase it in HTA parlance, are the cost of dossier requirements in line with their expected benefit of informing a better pricing decision? A buyer who commissions an audit of 30,000 pages before accepting a price quote is not a more rational buyer but a dysfunctional one. The pharmaceutical pricing transaction is no different in principle, even if it differs in social stakes and institutional complexity. If 30,000 patients are genuinely needed to report consistently on the relevant PICOs and subgroups, that is itself relevant information.

## 3. Access Conditions Are Price Instruments

A critical analytical move, often elided in HTA discourse, is the recognition that access conditions of every variety are ultimately expressions of price tension, the gap between the price demanded by the manufacturer and the price the payer considers acceptable given the evidence and its own legitimate budgetary constraints. This tension is not a malfunction but the appropriate response to market failure.

Therapeutic restriction to a subpopulation, the most common form of conditioned access in European systems, reflects one of two pricing logics [16]. In the first, the payer restricts access to the subgroup in which the product achieves cost-effectiveness at the requested price. In the second, the payer restricts access to the subgroup where clinical benefit is largest, reflecting the reluctance to pay for marginal benefit at a price calibrated to a broader population. In both cases, the restriction is a price instrument: it narrows the scope of access until the product’s benefit-to-price ratio becomes acceptable within that scope (which is fully appropriate). Restriction attributable purely to clinical concerns does occur, but it is the exception rather than the rule. In Germany, even subpopulations without demonstrated additional benefit can be used to exert downward pressure on the reimbursement price.

Managed entry agreements (MEAs) function similarly. Financial-based MEAs, confidential rebates, price-volume arrangements, and payment caps directly reduce the effective net price, either by returning a percentage of revenue, by capping expenditure when patient volumes exceed a threshold, or by establishing a ceiling on total budget impact [17]. Performance-based agreements, outcomes-based contracts, coverage with evidence development, and conditional reimbursements are more complex in design but equally price-oriented in function: they align the price on observed performance, reducing the payer’s exposure to uncertainty about clinical benefit and implicitly reducing effective net price in scenarios where outcomes fall below projected thresholds [18]. Even price-volume agreements, which ostensibly manage budget impact rather than set unit prices, function as instruments of fiscal exposure control; they are price discounts expressed in volumetric rather than unit terms.

This taxonomy of access conditions as price instruments is not a theoretical abstraction. It is the working logic of every national pricing body in Europe. Understanding it clarifies that the purpose of the buyer’s specification audit, HTA, is to supply the evidence needed to calibrate these instruments to an appropriate price, not to produce a definitive scientific verdict on the product’s place in medicine. It serves rather to specify efficacy and safety relative to therapeutic alternatives so as to inform pricing and reimbursement.

Tainter described how complex societies progressively invest in administrative and infrastructural structures to solve their problems. Each solution generates new problems requiring yet more complexity to resolve them. The marginal returns on this investment in complexity inevitably diminish, creating a deficit. When this deficit becomes unsustainable, the society can no longer finance its own complexity and collapses rapidly. Collapse is therefore not an irrational catastrophe [19].

## 4. The EU-HTA Proportionality Failure

EU Regulation 2021/2282 created the JCA as a mandatory pan-European clinical assessment for oncology and advanced therapy medicinal products, with phased extension to other therapeutic areas [12]. The political logic was sound in ambition: eliminate duplicative national assessments, reduce the burden on manufacturers, accelerate patient access, and improve consistency across member states [20]. In practice, the architecture has produced an assessment process of extraordinary procedural weight.

The evidentiary requirements for JCA dossiers have proven, in early experience, to substantially exceed even the most demanding national HTA submissions. At least one submission has reportedly required documentation approaching 30,000 pages. Standard submissions routinely achieve several thousand pages. The PICO proliferation problem, where the assessment body generates 10 to 23 distinct PICO frameworks for a single product where one to three were anticipated, compounds this burden, requiring manufacturers to supply evidence across a multiplicity of comparators, populations, subgroups, and outcome sets that may have limited clinical relevance [21,22,23,24]. The procedural timeline adds further complexity, with JCA publication now preceding national pricing processes, meaning that member states receive a clinical assessment that they may accept, reinterpret, or effectively set aside, followed by a national process that reassembles much of the same evidence for local purposes.

The result is a tripartite assessment structure that is without precedent in commercial transaction logic: a European-level clinical assessment, a national HTA process (retained, in full, by all member states), and a national pricing committee process. The buyer is conducting three sequential specification audits, each substantial in scope, before agreeing to a price. The costs of this architecture, financial, temporal, and organizational, are not trivial, and they are borne asymmetrically, with smaller manufacturers facing disproportionate burdens relative to their capacity. In effect, the architecture favors large multinationals over start-ups and SMEs, raising concerns for the competitiveness of Europe’s pharmaceutical sector.

It is important to note that the pharmaceutical pricing transaction occurs in vastly more complex commercial environments every day without equivalent procedural weight. States negotiate defense procurement contracts for technically intricate products without 30,000-page specification audits. Healthcare systems procure large apparatuses such as those for imaging, complex medical devices, and surgical technologies through processes that, while rigorous, are calibrated to the decision at hand. The JCA architecture is, in comparative terms, a procedural outlier, and the outlier status is not justified by the nature of the transaction it is designed to support.

## 5. Value-Based Pricing, Uncertainty, and Proportionate Evidence Requirements

The European pharmaceutical pricing model is built on value-based principles, modulated by uncertainty and affordability [25,26,27]. The price is expected to reflect the incremental therapeutic benefit of the product relative to the standard of care, adjusted for the payer’s willingness to pay and for the uncertainty surrounding the evidence base. This is a well-developed framework; the German AMNOG system, the French ASMR architecture, and NICE’s probabilistic cost-effectiveness modeling are all operationalized versions of it [4,6,7].

The implications for the JCA are important. If the purpose of the JCA is to establish the evidence base for value-based pricing, then the depth of assessment should be proportionate to the degree of uncertainty that is actually decision-relevant, that is, uncertainty that materially affects the price that member states should be willing to pay. Extensive documentation of exhaustive characterization of adverse events in populations that will not be treated and PICO proliferation across comparators that have no market presence in most member states do not reduce decision-relevant uncertainty. They increase documentation volume without improving transaction quality.

The EU Joint Clinical Assessment operates under a tacit but consequential misconception: that scientific rigor is proportional to documentary volume, that quality is synonymous with quantity, and that exhaustiveness constitutes fitness for purpose. These conflations are not merely procedural and administrative inefficiencies; they represent a structural category error. Rigor is a property of methodology, of the validity of comparators selected, of the appropriateness of endpoints chosen, and of the transparency of uncertainty characterized, not of the weight of evidence accumulated [28]. Exhaustiveness, taken to its logical extreme, becomes its own form of analytical failure: when every subgroup is assessed, every comparator included, and every sensitivity analysis documented regardless of decision relevance, the signal is buried in noise, and the assessment loses its capacity to inform a decision. Fitness for purpose demands the opposite discipline, the deliberate selection of evidence that is sufficient, not maximal. Such selection requires value judgements that the assessment body has been reluctant to assume. EU-HTA, by confusing these categories, has produced a framework that is simultaneously over-engineered and under-targeted: it demands more than any national healthcare system has ever required, yet delivers an output that member states must largely reprocess for their own pricing purposes. The result is an assessment architecture that exhausts without informing, documents without deciding, and mistakes procedural weight for scientific authority. The JCA has mutated into a self-referential bureaucratic exercise, driven by procedural complexity metastasizing until the report displaces the purpose it was meant to serve.

An instructive historical parallel is provided by Hans Christian Joachim Gram (1853–1938), a Danish bacteriologist and physician whose landmark paper, published in 1884, described a staining method in only a few pages [29]. The Gram stain remains today one of the most consequential diagnostic tools in the history of microbiology. Scientific consequence, then as now, is a function of methodological clarity and decision relevance, not of documentary volume.

A proportionate JCA would ask a more focused question: What is the minimum credible evidence base needed to support a reliable assessment of incremental clinical benefit in the relevant patient population, against the relevant comparator, and the level of uncertainty that will inform the pricing arrangements that will follow? That question has a manageable answer, one that does not require several thousand pages.

## 6. Conclusions

HTA is reimbursed price determination. Every access condition, restriction, financial MEA, performance-based contract, price-volume arrangement, and expenditure cap is an expression of price tension between what a manufacturer requests and what a payer accepts. The buyer’s audit of product specification has a legitimate and important role in pharmaceutical governance, but it should be proportionate to the transaction it serves. The JCA is not the end of a process but just the beginning. It should be actionable to serve its purpose.

EU-HTA, in its current JCA architecture, has produced a procedural bureaucratic framework that is structurally disconnected from this transactional logic. The complexity is not justified by the decision-relevant uncertainty it addresses; the tripartite assessment structure (European JCA, national HTA, and national pricing committee) imposes a redundant burden on all parties; and the PICO proliferation problem compounds the issue by expanding the scope of assessment beyond what member states will use or require. The current JCA reporting can be considered not fit for purpose.

Reform of EU-HTA requires a frank confrontation with first principles. The question is not ‘how thorough should the assessment be?’ but ‘what does the pricing transaction actually need?’ Answering the right question is the precondition for building a proportionate system. European patients, payers, and the pharmaceutical industry as innovation developers deserve one.

## Data Availability

No new data were created or analyzed in this study. Data sharing is not applicable to this article.

## Abbreviations

The following abbreviations are used in this manuscript:

AMNOG	Arzneimittelmarkt-Neuordnungsgesetz (German Act on the Reform of the Market for Medicinal Products)
ASMR	Amélioration du Service Médical Rendu (improvement in actual benefit)
CEPS	Comité Économique des Produits de Santé (Economic Committee for Health Products)
EU-HTA	European Union health technology assessment
G-BA	Gemeinsamer Bundesausschuss (Federal Joint Committee)
GKV-Spitzenverband	National Association of Statutory Health Insurance Funds
HAS	Haute Autorité de Santé (French National Authority for Health)
HTA	health technology assessment
JCA	joint clinical assessment
MEA	managed entry agreement
NICE	National Institute for Health and Care Excellence
PICO	population, intervention, comparator, outcome
QALY	quality-adjusted life year
RCT	randomized controlled trial
SME	small- and medium-sized enterprise
